# Jejunal obstruction due to rare internal hernia between skeletonized external iliac artery and vein as late complication of laparoscopic hysterectomy with pelvic lymphadenectomy—case report and review of literature

**DOI:** 10.1007/s00404-020-05724-x

**Published:** 2020-08-07

**Authors:** Felix Frenzel, Sebastian Hollaender, Peter Fries, Russalina Stroeder, Jonas Stroeder

**Affiliations:** 1 Clinic for Diagnostic and Interventional Radiology, Saarland University Medical Center, 66421 Homburg, Saar Germany; 2 Clinic for General, Abdominal and Vascular Surgery, Saarland University Medical Center, 66421 Homburg, Saar Germany; 3 Clinic for Gynecology and Obstetrics, Saarland University Medical Center, 66421 Homburg , Saar Germany

**Keywords:** Internal hernia, Lymphadenectomy, Small bowel obstruction, Iliac vessels, Case report

## Abstract

**Background:**

Internal herniation of small intestine in the lesser pelvis alongside iliac vasculature is a rare occurrence. Skeletonization of iliac vessels during pelvic lymph node dissection (LND), as part of surgical staging or treatment of patients with uterine, ovarian or urogenital cancer, is a strict prerequisite for orifice formation.

**Case presentation:**

A 68-year-old woman presented at the emergency department with complaints of constipation for the last 3 days and acute-onset abdominal pain, nausea and vomiting since few hours. She had a history of laparoscopic hysterectomy, bilateral salpingo-oophorectomy and para-aortic and pelvic LND 7 years ago. A distended abdomen with diffuse tenderness on palpation was noted. A CT scan demonstrated bowel obstruction secondary to an incarcerated hernia underneath an elongated right external iliac artery. During an emergency exploratory laparotomy, the incarcerated bowel was reduced and the hernial orifice closed with a running suture. The patient had an uneventful postoperative period and was discharged on the fifth postoperative day.

**Discussion:**

This rare internal hernia can manifest with non-specific symptoms of small bowel obstruction at any given point after index surgery, sometimes even after several years free of complaints. Contrast-enhanced computed tomography is the method of choice for fast and reliable diagnosis and helps in planning the necessary emergency laparotomy.

**Conclusion:**

This life-threatening complication adds to the current controversy of pelvic and para-aortic lymphadenectomy in patients with endometrial cancer. Primary closure of peritoneal defects should be considered to potentially prevent internal hernias, especially when elongated iliac vessels are present.

**Electronic supplementary material:**

The online version of this article (10.1007/s00404-020-05724-x) contains supplementary material, which is available to authorized users.

## Introduction

Internal hernias originate from passage of intestinal structures, usually small intestine, through congenital or acquired apertures within the boundaries of the peritoneal cavity. Clinical complaints result from obstruction of intestinal loops, which may end up necrotic due to incarceration if left untreated. Similar to external hernias, they oftentimes present with signs of severe passage disorder: acute or subacute onset abdominal pain, nausea and vomiting as far as fecal vomiting. However, they can as well remain asymptomatic for a long time or present with intermittent or constant vague epigastric pain. Left paraduodenal herniation of jejunal loops through Landzert’s fossa is the most common form of internal hernia [1].

Herniation of small intestinal loops in the lesser pelvis alongside the iliac vessels is a rare entity that has only been reported in eight cases in English-language literature today (Table 1). Removal of retroperitoneally located iliac vessels’ peritoneal coverage (“skeletonization”) is the prerequisite for hernial orifice formation and a consequence of pelvic lymphadenectomy (LND). The dissection of pelvic and para-aortic lymph nodes is a standard procedure in many operative cancer treatments and common in prostate, bladder, ovarian or cervical cancer. Small bowel obstruction (SBO) due to herniation underneath an exposed common iliac artery (CIA) was initially reported in 1978 following open pelvic LND during treatment of testicular teratocarcinoma [2]. Except for open LND in one patient with ovarian cancer, three further patients had a history of laparoscopic pelvic LND for treatment of cervical cancer, while three patients underwent robot-assisted pelvic LND for urological malignancies. Herniation was reported underneath external or common iliac artery as well as the space between right obturator nerve and umbilical artery in one patient.

**Table 1:** Reported cases of small bowel obstruction caused by vessels after pelvic lymphadenectomy in English-language literature

Patient	Cancer	Index operation	Operative approach	Obstructing vessel	latency^a^	Treatment of obstruction	Author	Year
52 years, male	Testicular terato-carincoma	Radical retroperitoneal LND, periaortic and inguinal irradiation	Open	Right CIA	4 months	Open bowel resection (ileum), ileostomy, closure of orifice with free peritoneal graft	Guba et al. [2]	1978
67 years, female	Cervical cancer	Extended hysterectomy and pelvic LND	Laparoscopic	Right EIA	3 months	Open bowel resection and peritoneal closure	Kim et al. [5]	2008
56 years, female	Serous papillary adeno-carcinoma (ovary)	Total abdominal hysterectomy, omentectomy, appendectomy, radical retroperitoneal LND	Open	Left EIA	4 years	Laparoscopic release of bowel obstruction, no bowel resection or orifice repair	Dumont et al. [15]	2013
39 years, female	Cervical carcinoma	RVT with pelvic LND (Dargent's operation)	Laparoscopic	Right CIA	2 years	Open bowel resection (ileum) with primary side-to-side anastomosis, orifice closure with collagen patch	Ardelt et al. [16]	2014
50 years, male	Squamous cell carcinoma (bladder)	Partial cystectomy and ePLND	Robot-assisted	Right CIA	5 months	Open bowel resection (ileum), cover of iliac artery with peritoneal flaps	Pridjian et al. [17]	2015
50 years, male	Prostate cancer	Radical prostatectomy and ePLND, radiotherapy of prostatic bed and left iliacal axis	Robot-assisted	Left EIA (elongated)	1 year	Open bowel resection, resection of elongated EIA (both with end-to-end anastomosis), fixation of artery to lateral peritoneum, fibrin sealant patch	Viktorin-Baier et al. [4]	2016
64 years, male	Prostate cancer	Prostatectomy with ePLND	Robot-assisted	Right EIA	1 year	Open bowel resection (ileum) with primary anastomosis	Kambiz et al. [18]	2018
38 years, female	Cervical cancer	Extended hysterectomy and pelvic LND	Laparoscopic	Space between right obturator nerve and umbilical artery	6 months	Laparoscopic release of bowel obstruction, resection of umbilical artery	Minami et al. [19]	2018
68 years, female	Endometrial adeno-carcinoma	H-BSO, pelvic and para-aortic LND, adjuvant vaginal brachytherapy	Laparoscopic	Between right EIA and EIV	7 years	Open bowel resection (ileum) with primary end-to-end anastomosis, orifice closure with running suture	This paper	2020

*CIA* common iliac artery, *EIA* external iliac artery, *EIV* external iliac vein, *LND* lymph node dissection, *ePLND* extended pelvic lymph node dissection, *RVT* radical vaginal trachelectomy, *H-BSO* hysterectomy and bilateral salpingo-oophorectomy

^**a**^Latency meaning time between index operation and onset of symptoms

Latency between surgery and onset of abdominal symptoms was variable in all prior cases published, ranging from 3 months to 4 years. We report the first case of a patient who underwent surgery for endometrial cancer, had an unusually long latency until onset of symptoms and featured a yet unreported orifice of herniation between the external iliac artery (EIA) and vein (EIV).

## Case summary

A 68-year-old severely obese lady (BMI 37,6 kg/m^2^) presented with a few-hours history of acute-onset abdominal pain, nausea and vomiting in our clinic’s internal medicine emergency department. She reported symptoms of constipation for 3 days prior to arriving at our clinic. Her medical and surgical history was empty apart from treatment of endometrioid endometrial adenocarcinoma with deep myometrial invasion (pT1b pN0 (0/30), L0, V0, R0; FIGO IB; Grade 2). She had undergone laparoscopic hysterectomy with bilateral salpingo-oophorectomy (H-BSO), para-aortic and pelvic LND as well as adjuvant vaginal brachytherapy over 3 weeks (7 Gy, 5 Gy, 5 Gy) 7 years ago in 2011. There was no abdominal surgery of any cause or abdominal complaints in the meantime.

Clinical examination revealed a distended abdomen with diffuse tenderness on palpation of the hypogastric region and slight muscular guarding. Initial abdominal X-ray revealed no classical signs of mechanical ileus. An abdominal sonography reported no pathological findings. Lab test indicated systemic infection with raised C-reactive protein (62 mg/l) and procalcitonin levels (0.1 ng/ml) as well as elevated leukocyte count (13.8 × 10^3^/µl). First of all, antibiotic treatment for nitrite negative urinary tract infection was initiated due to leukocyturia (500/µl) and microhematuria in urine strip testing. A return-flow enema was performed due to the reported constipation.

Because of inflammatory markers continuing to rise (CRP 214 mg/l; PCT 0.45 ng/ml) under antibiotic therapy and in absence of pain relief despite the enema, a diagnostic contrast-enhanced computed tomography (CT) of the abdomen was performed the next day (Online Resource 1).

**Fig. 1:**
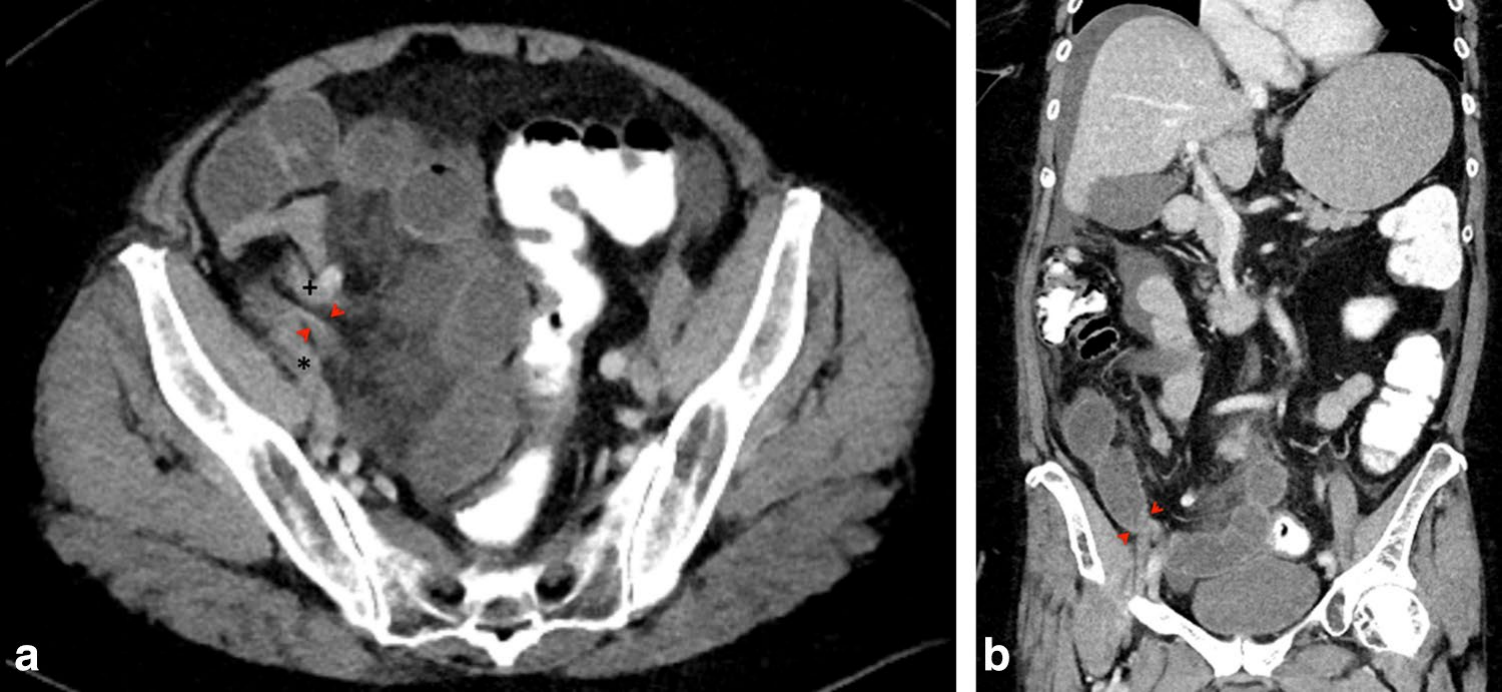
CT scan. **a** Axial view showing herniation of ileum with abrupt reduction of caliber (arrowheads) between external iliac artery ( +) and external iliac vein (*). **b** Coronal view, showing the site of herniation (arrowheads). Distension of proximal jejunal loops due to small bowel obstruction. Perihepatic and inter-enteric ascites in the right hemiabdomen as secondary finding

CT revealed mechanical SBO in the lower right abdomen with significant dilation, wall edema and contrast-enhancement of distal loops of jejunum and proximal loops of ileum. The cause was identified to be a herniation of the proximal ileum between the EIA and EIV in the right iliac fossa with consecutive significant stenosis of the bowel lumen, abrupt change of caliber and total collapse of the terminal ileum (Fig. 1). Strikingly, the right hemiabdomen showed a “dirty fat” sign with extensive streaky infiltration of visceral fat and enlarged lymph nodes as well as significant amounts of diffuse ascites with emphasis on the right paracolic gutter, caecum and perihepatic space. This raised suspicion of a generalized peritonitis, showing inflammatory involvement of the right ureter and giving an explanation to the positive urine test strip.

**Fig. 2:**
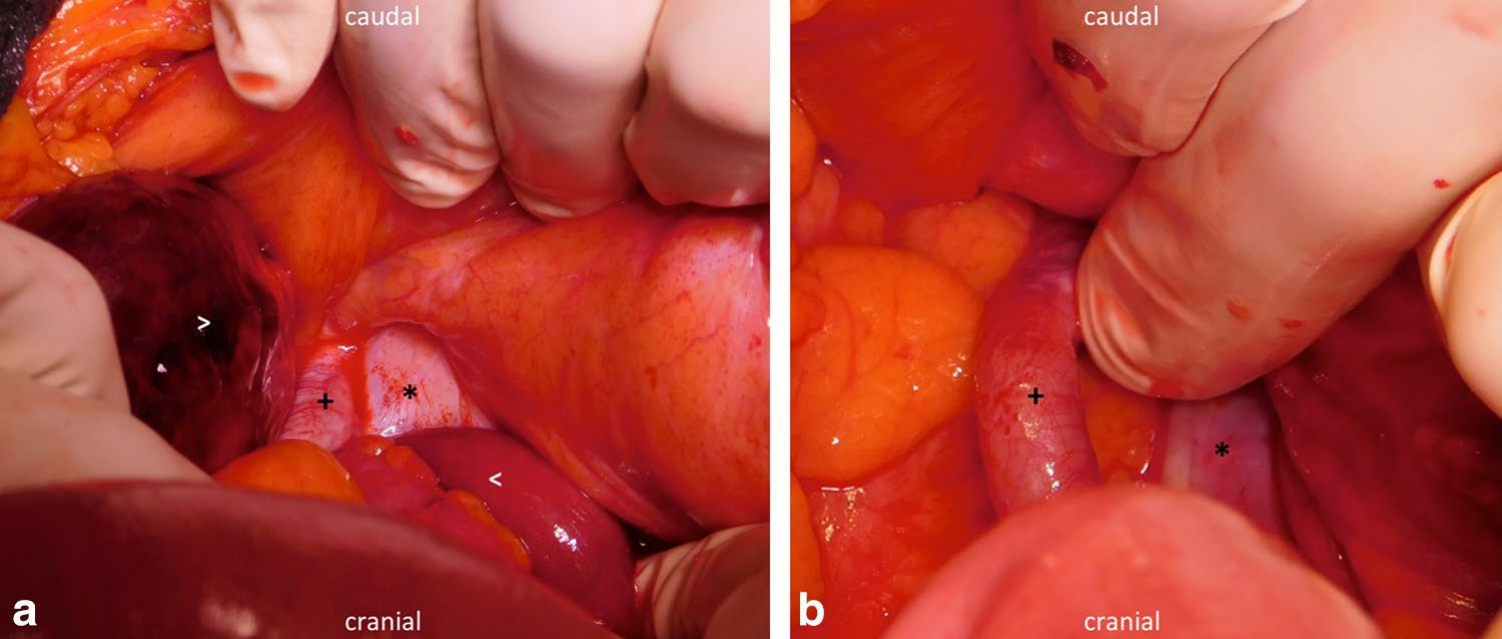
Intraoperative sight. **a** Ileum proximal ( <) to herniation between the external iliac artery ( +) and external iliac vein (*). The herniated ileum loop ( >) presents clear signs of incarceration. **b** Demonstration of the hernial orifice between the external iliac artery ( +) and external iliac vein (*) after reposition and resection of the incarcerated ileum loop

The same day, the decision for exploratory laparotomy was made to confirm the radiologic findings. Intraoperatively, the parietal peritoneum presented macroscopically reddened and showed vascular injections in the right upper and lower abdomen. A conventional abdominal lavage using 10 L of Ringer’s lactate solution was performed to remove ubiquitous cloudy ascites and to decrease bacterial counts in diluted peritoneal fluid. Inspection of the right iliac fossa revealed a distended, gangrenous ileum loop caused by incarceration due to an internal herniation of the ileum between the skeletonized EIA and EIV (Fig. 2). Thorough inspection of the small intestine revealed no signs of perforation. There were no adhesions after former surgery that might have facilitated the herniation and no signs of actinic enteritis that would mimic the diagnosis. However, the ipsi- and contralateral iliac sites of lymphadenectomy showed striking anatomical differences. The right EIA was distinctly elongated and formed an arch, creating a significant free space between EIA and normal EIV which allowed the intestinal loop to become trapped underneath the artery. On the left side, the iliac vessels did not show any anatomical abnormalities. Left EIA and EIV were running side-by-side and their adventitia did adhere, creating no preformed herniation orifice.

The incarcerated loop was removed from the hernial orifice and a segmental resection of 50 cm ileum was performed approximately 200 cm distally to the ligament of Treitz with primary side-to-side jejuno-ileal anastomosis. A running suture with resolvable suture material (PDS Gauge 5–0) was used for primary closure of the hernial orifice to prevent future re-herniation.

The patient’s aftercare was uneventful with rapid recovery of abdominal symptoms and inflammatory parameters. She was discharged 5 days after surgery in good general condition with normal bowel activity and antibiotic treatment (Ceftriaxone/Metronidazole) completed 8 days after surgery.

## Discussion

The most common cause of postoperative bowel obstruction is adhesions. Different forms of internal or abdominal wall hernias are much rarer conditions, each with a challenging diagnosis and high mortality rate, exceeding 50 % in some series [1]. Regarding our case’s clinical and anatomical features, an appropriate but rare differential diagnosis to an adhesive ileus is the obturator hernia. This “little old lady’s hernia” occurs especially in elderly female and post-pregnancy patients (such as the average population submitted to pelvic LND) with recent weight loss (similarly to the loss of fatty tissue during LND) [3].

Strangulation of small bowel loops by an external or common iliac artery is an even rarer, but nevertheless important differential diagnosis for ileus in patients with history of surgical procedures that involve pelvic LND. The removal of lymphatic tissue and the resulting defect in the peritoneum frequently create a significant free space between the arching retroperitoneal vessels, which is increased even further in patients with severe kinking and elongation of pelvic arteries [4]. Several authors showed different approaches to prevent recurrence of internal hernias using free peritoneal grafts, collagen patches or fibrin sealants to cover exposed vessels (Table 1). While transperitoneal LND is a standard procedure during surgical treatment of urogenital cancer, there are no established guidelines or recommendations regarding primary closure or non-closure of the peritoneum to prevent internal hernias. In spite of theoretical advantages, preventive peritoneal closure after pelvic LND is uncommon. In cervical and endometrial cancer, there has been no difference in postoperative morbidity for closure vs. non-closure [4]. Still, covering of exposed pelvic vasculature should at least be discussed in patients with tortuous, elongated iliac arteries, which could be causal lesions of an internal hernia [4, 5].

Furthermore, this rare complication adds to the controversy of pelvic and para-aortic LND as part of the surgical staging in patients with endometrial cancer, as recommended by the “Fédération Internationale de Gynécologie et d’Obstétrique” (FIGO) since 1988. According to their 2018 cancer report, as a minimum, any enlarged or suspicious lymph nodes should be removed in all patients. For high-risk patients (grade 3, deep myometrial invasion, cervical extension, serous or clear cell histology), complete pelvic LND and resection of any enlarged para-aortic nodes is recommended [6]**.** Lymphatic metastases are one of the most important prognostic factors and LND may be beneficial by identifying lymph node positive patients in need of adjuvant treatment, including radiotherapy and chemotherapy. However, over 30 years since their first recommendation by the FIGO, the role of systematic lymph node staging, as well as its therapeutic benefits, remain highly controversial. In 2008–2009, findings of two large independent randomized trials suggested that pelvic LND merely adds increased morbidity without unambiguous influence on survival outcomes [7, 8]. In 2017, a review by Frost et al. showed no benefit for LND in overall and recurrence-free survival but a significantly higher risk of surgery-related systemic morbidity and lymphedema or lymphocyst formation [9]. While LND may even clear metastases, lymphatic dissemination is relatively uncommon in endometrial cancer, so in theory only the minority of patients should benefit from LND. This hypothesis is supported by a current study by Ignatov et al., who showed that systematic pelvic and para-aortic LND did not improve the survival of patients with early stage I and II endometrioid (type I) endometrial cancer at intermediate and high risk of recurrence [10]. In women undergoing H-BSO and pelvic LND for type I endometrial cancer, two independent studies from Li et al. and Pauly et al. in 2020 found no improvement of surgical outcomes or prognosis following an additional para-aortic LND [11, 12], suggesting that the extent of LND does not impact the disease-specific survival. In patients with type I endometrial cancer, pelvic LND alone might be sufficient from a diagnostic perspective because skip metastases in para-aortic lymph nodes are rarely seen [11].

However, in patients with early-stage non-endometrioid (type II) endometrial carcinoma and carcinosarcoma which tend to metastasize in pelvic and para-aortic lymph nodes more frequently, systematic pelvic and para-aortic LND independently and significantly prolonged the patients’ survival [13].

The concern about a potential overtreatment of many patients (especially those with type I endometrial cancer), who oftentimes are burdened with an adverse surgical risk profile due to obesity, old age and other co-morbidities led to sentinel node mapping being increasingly utilized for endometrial cancer staging in the recent years  [14].

In 2019, a meta-analysis of six comparative studies displayed non-inferiority of sentinel node mapping to standard LND in detection of para-aortic nodal involvement and recurrence rates while even suggesting a possible superiority in detection of pelvic lymph node involvement [14]. Anticipated further randomized studies will allow to asses this method’s long-term effectiveness.

Right now, systematic pelvic and para-aortic LND is recommended for high-risk patients (FIGO Stage ≥ IB, Grade 3). In our case, it was performed for Grade 2 endometrioid endometrial adenocarcinoma (FIGO IB), where it is neither absolutely necessary nor dissuaded.

## Conclusion

SBO due to herniation beneath skeletonized pelvic vasculature is an odd complication that further adds to the morbidity of patients after pelvic LND. This uncommon complication may be considered as differential diagnosis in patients who present with signs of bowel obstruction and have a positive history of lower pelvic surgery including operative treatment of prostate, bladder, ovarian, cervical or endometrial cancer. The latency between the index operation and symptomatic herniation shows a wide range of variation according to former case reports and may be even misleadingly long, as our case demonstrates. This circumstance reinforces the weight of a thorough and reflective anamnesis allowing for a shortened diagnostic process and urgent surgical intervention, which both are imperative to treating patients with this particular complication. While this hernia was initially overlooked in our patient in an ultrasound examination due to its uncommon location, contrast-enhanced computed tomography, being the diagnostic method of choice, showed unmistakable results.

## Takeaway message

Clinicians should be aware of the existence of rare iatrogenic hernias through artificial orifices due to the inevitable separation of pelvic vessels during lymphadenectomy.

## Electronic supplementary material

Below is the link to the electronic supplementary material.

Supplementary file1 (MP4 18210 kb) Online Resource 1 – CT scan (video) Contrast-enhanced CT-Scan performed on a Somatom Definition Edge Single Source CT Scanner (Siemens Healthineers) in a portal-venous phase using a bolus-tracking technique after i.v. contrast agent injection (Imeron® 400, Bracco Imaging); axial orientation. There is evidence of a mechanical SBO in the lower right abdomen with significant dilation of the upper GI-tract including a massively fluid-filled stomach. In the right iliac fossa, a herniation of the proximal ileum between the elongated, arching EIA and EIV can be seen with an abrupt change of caliber and total collapse of the terminal ileum. An extensive streaky infiltration of visceral fat, enlarged lymph nodes and significant amounts of diffuse ascites can be detected with emphasis on the right paracolic gutter, caecum and perihepatic space, consistent with the diagnosis of peritonitis.
